# Social Vocalizations of Big Brown Bats Vary with Behavioral Context

**DOI:** 10.1371/journal.pone.0044550

**Published:** 2012-09-07

**Authors:** Marie A. Gadziola, Jasmine M. S. Grimsley, Paul A. Faure, Jeffrey J. Wenstrup

**Affiliations:** 1 Department of Anatomy and Neurobiology, Northeast Ohio Medical University, Rootstown, Ohio, United States of America; 2 School of Biomedical Sciences, Kent State University, Kent, Ohio, United States of America; 3 Department of Psychology, Neuroscience & Behaviour, McMaster University, Hamilton, Ontario, Canada; University of Southern California, United States of America

## Abstract

Bats are among the most gregarious and vocal mammals, with some species demonstrating a diverse repertoire of syllables under a variety of behavioral contexts. Despite extensive characterization of big brown bat (*Eptesicus fuscus*) biosonar signals, there have been no detailed studies of adult social vocalizations. We recorded and analyzed social vocalizations and associated behaviors of captive big brown bats under four behavioral contexts: low aggression, medium aggression, high aggression, and appeasement. Even limited to these contexts, big brown bats possess a rich repertoire of social vocalizations, with 18 distinct syllable types automatically classified using a spectrogram cross-correlation procedure. For each behavioral context, we describe vocalizations in terms of syllable acoustics, temporal emission patterns, and typical syllable sequences. Emotion-related acoustic cues are evident within the call structure by context-specific syllable types or variations in the temporal emission pattern. We designed a paradigm that could evoke aggressive vocalizations while monitoring heart rate as an objective measure of internal physiological state. Changes in the magnitude and duration of elevated heart rate scaled to the level of evoked aggression, confirming the behavioral state classifications assessed by vocalizations and behavioral displays. These results reveal a complex acoustic communication system among big brown bats in which acoustic cues and call structure signal the emotional state of a caller.

## Introduction

Acoustic communication plays a primary role in social interactions among many species of bats. Social vocalizations are context-specific [1], [2], [3], [4], [5], [6] and carry significant information about the caller, including identity of individuals [7], [8], [9], [10], [11], [12]. The spectro-temporal features of bat social vocalizations are highly diverse and can be combined in elaborate sequences, such as complex mating songs [13], [14], [15]. Moreover, complex social vocalizations are learned in some species of bats [16], [17], [18], [19], including babbling behavior in bat juveniles [20]. Although such acoustic phenomena are widespread among songbirds, the complexity of acoustic communication systems observed in bats are exceptional in the mammalian world, making bats an important model for studies of acoustic communication.

This study examines how vocalizing animals convey information about their affective or emotional state in social interactions. Recent work has focused on how syllable acoustics, temporal emission patterns, and call composition vary within vocalizations to signal the behavioral context [21]. Although the behavioral context of Mexican free-tailed bat (*Tadarida brasiliensis*) vocalizations can often be distinguished by unique syllabic acoustics, some syllables are common across contexts but are emitted in distinct temporal patterns [21]. Further, the intensity level of an affective state may be effectively encoded by graded changes in acoustic features, such as the repetition rate [22]. Evidence of graded acoustic parameter changes are widespread across mammals, suggesting a common coding rule in mammalian communication sounds [23], [24], [25], [26], [27], [28]. In bats, there is recent evidence that greater false vampire bats (*Megaderma lyra*) make systematic changes to the call structure, such as the number and repetition rate of syllables, according to the intensity level of agonistic interactions [9], and such changes provide prosodic cues that listeners may evaluate [29]. Nonetheless, important questions still surround the accuracy with which the acoustic signals reflect the internal affective state of the caller and what influence these sounds have on a listener.

Big brown bats (*E. fuscus*), the focus of the current study, are among the most abundant and widespread colonial bats in North America [30]. Their biosonar signals and associated processing by the auditory system have been studied extensively [31], [32], [33]. However, with the exception of mother-infant interactions [25], [34], [35], there have been no detailed reports on the repertoire of adult social communication calls in the big brown bat or on the extent to which social calls convey information about emotional state. This information is particularly needed in light of recent work showing that neurons in the big brown bat amygdala, a center of emotional processing, discriminate well among syllables of social vocalizations [36]. An improved description of the vocal repertoire, associated behavioral contexts, and emotional states will facilitate a better understanding of the roles of brain regions involved in acoustic communication.

In the present study we identified acoustic features of adult social vocalizations of captive big brown bats and related these to behavioral contexts. We show that big brown bats have a rich repertoire of social vocalizations, identifying 18 syllables with unique spectral-temporal features that were automatically classified using a spectrogram cross-correlation procedure. Emotion-related acoustic cues were found within the call structure in both syllable composition and temporal emission pattern. Further, we found that heart rate scaled to the level of evoked vocal aggression, confirming the behavioral state classifications assessed by vocalizations and behavioral displays.

## Methods

### Ethics Statement

Animal husbandry and experimental procedures were approved by the Institutional Animal Care and Use Committees at Northeastern Ohio Medical University (NEOMED) (Protocol number 10-001) and the McMaster University Animal Research Ethics Board (Protocol number 08-07-34), and were performed in accordance with guidelines published by the U.S. National Institutes of Health and the Canadian Council on Animal Care.

### Acquisition and Maintenance of Animals

Social vocalizations were recorded from big brown bats (*E. fuscus*) maintained in captive research colonies at McMaster University and NEOMED. Vocalizations were recorded from 10 adult bats (6 females, 4 males) from the McMaster colony. These bats were housed in a free-flight husbandry room (2.5×1.5×2.3 m) where temperature and lighting varied according to ambient conditions, with access to an outdoor flying area (2.5×3.8×3.1 m). Bats were given *ad libitum* access to water and mealworms.

Vocalizations were also recorded from 18 adult (14 females, 4 males) and three male juvenile bats from the NEOMED colony, some of which (n = 9) were recorded immediately after capture from the wild. These bats were housed in a free-flight husbandry room (2.6×4.6×2.6 m), and were given *ad libitum* access to water and mealworms.

**Figure 1:**
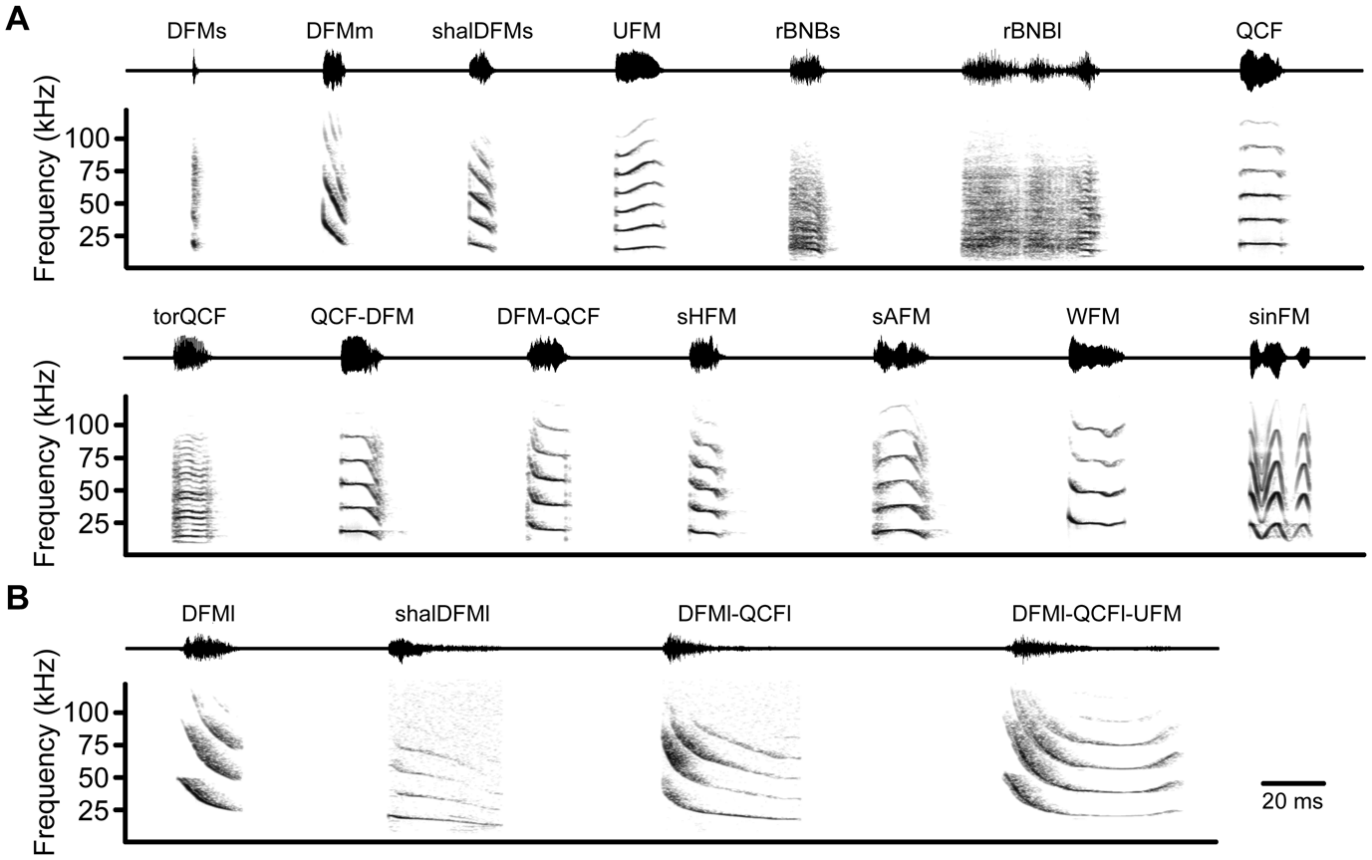
Syllable types of big brown bat social vocalizations. Example waveforms (*upper trace*) and spectrograms (*lower trace*) of 14 aggressive syllables (**A**), and 4 appeasement syllables (**B**). The time interval between syllables is for illustrative purposes and does not correspond to natural syllable emission rates. Peak amplitude of all syllables is normalized. *Abbreviations*: DFMs, downward frequency modulation, short; DFMm, downward frequency modulation, medium; shalDFMs, shallow downward frequency modulation, short; UFM, upward frequency modulation; rBNBs, rectangular broadband noise burst, short; rBNBl, rectangular broadband noise burst, long; QCF, quasi-constant frequency; torQCF, torus quasi-constant frequency; QCF-DFM, quasi-constant frequency to downward frequency modulation; DFM-QCF, downward frequency modulation to quasi-constant frequency; sHFM, single-humped frequency modulation; sAFM, single-arched frequency modulation; WFM, wrinkled frequency modulation; sinFM, sinusoidal frequency modulation; DFMl, downward frequency modulation, long; shalDFMl, shallow downward frequency modulation, long. DFMl-QCFl, downward frequency modulation long to quasi-constant frequency long; DFMl-QCFl-UFM, downward frequency modulation long to quasi-constant frequency long to upward frequency modulation.

### Acoustic Recordings

We recorded and analyzed social vocalizations and associated behaviors of captive bats in defined social interactions. The majority of recordings occurred during the summer when bats are most active. To optimize recording quality, 2–5 animals were housed in a small cage (35×33×28 cm) in a room lined with anechoic foam. Vocalizations were recorded using 2–3 ultrasonic condenser microphones (CM16/CMPA, Avisoft Bioacoustics, Berlin, Germany) connected to amplifiers and A/D converters (UltraSoundGate 416H, Avisoft Bioacoustics). The gain of each microphone was independently adjusted to optimize the signal-to-noise ratio of the recording. Acoustic signals were digitized at 250 kHz with 16-bit depth, and monitored in real time with RECORDER software (Version 5.1, Avisoft Bioacoustics). The room was dimly lit to allow video recording of behavior with a digital camcorder (Panasonic PV-GS150) or webcam synchronized with the vocal recordings.

**Figure 2:**
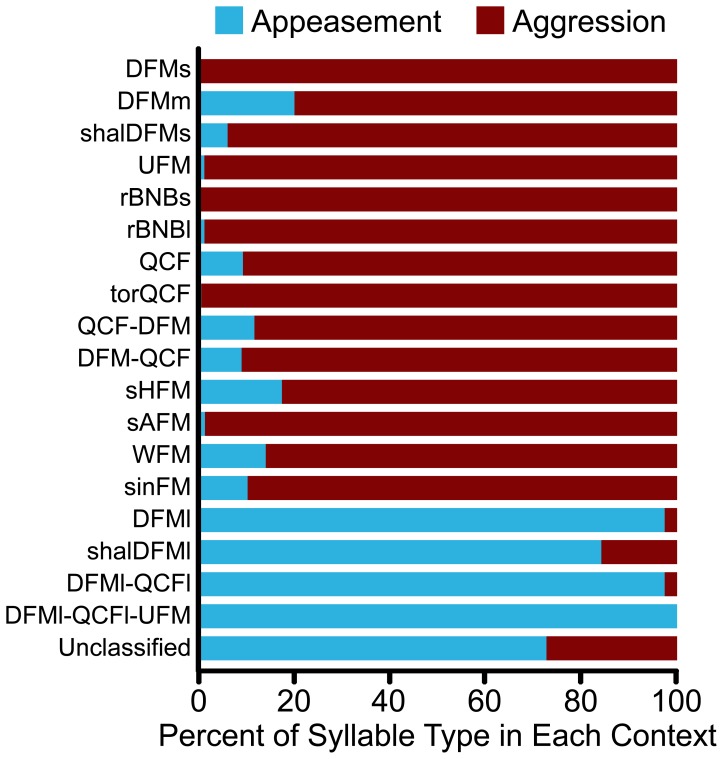
Occurrence of syllable types during aggression or appeasement. For all classified syllable types, more than 80% of observed syllables were emitted in a particular context, aggression (*red*) or appeasement (*blue*). We therefore classified these syllables as corresponding to the predominant behavioral context.

Roosting bats displayed limited movement and vocalizations. Each bat was banded for individual recognition. Similar to Clement et al. (2006), we recorded bats in a variety of paradigms to increase the probability of vocal emission:

In the *natural paradigm*, bats were undisturbed and recorded for several hours during their active period. Both aggressive and appeasing interactions were observed.

**Figure 3:**
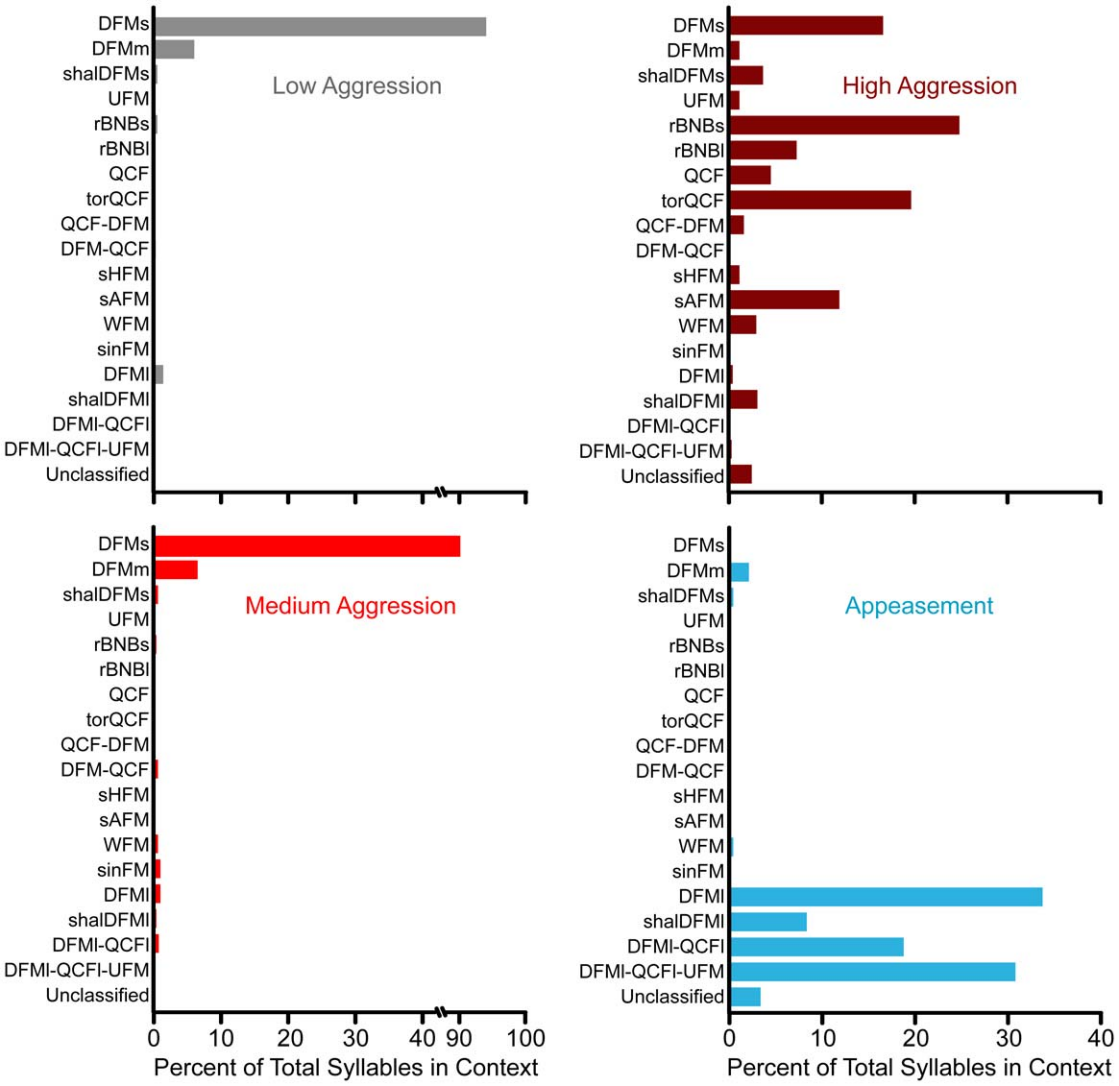
Probability of syllable types emitted across four behavioral contexts. The DFMs syllable type was the predominant syllable in both low aggression (*grey*) and medium aggression (*light red*) contexts. Syllable diversity increased during high aggression (*dark red*), with rBNBs being the most common syllable type. Four appeasement syllables were commonly emitted during appeasement (*blue*). Note the broken *horizontal axis* for low and medium aggression.

In the *intruder paradigm*, three days prior to recording, 3–4 bats were removed from the main colony and housed in a temporary “home-cage”. At the start of a trial, an “intruder”–another bat from the main colony–was introduced into the home-cage by the experimenter. Typically, the intruder crawled over and attempted to roost with the home-cage bats, often disturbing one as it jostled for a roost position within the group. This would often elicit aggressive displays and vocalizations from the disturbed bat, but not from the undisturbed home-cage bats. Recording sessions included several 10-min trials with different intruders.

**Figure 4:**
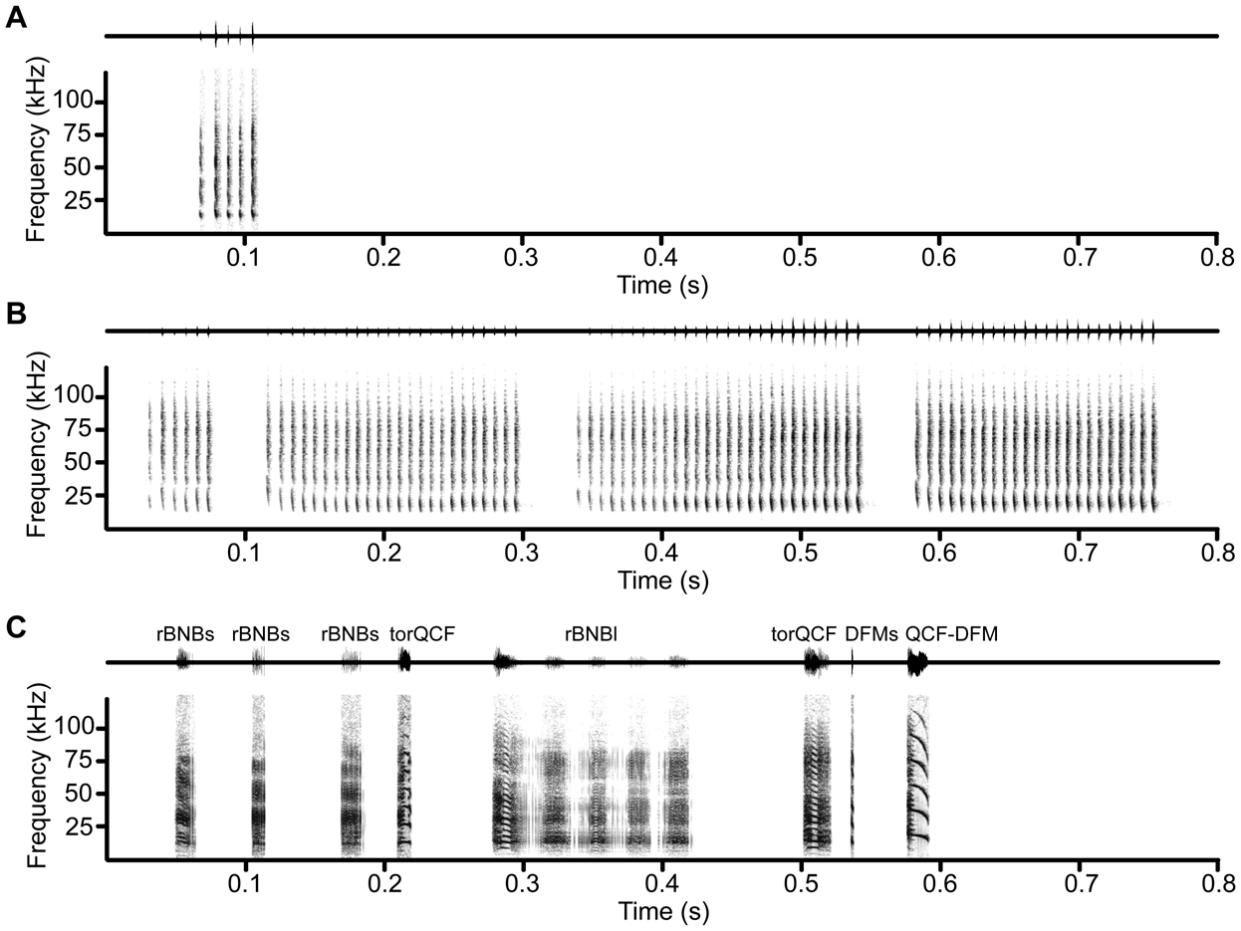
Example bouts of aggression vocalizations. Example waveforms (*upper trace*) and spectrograms (*lower trace*) of example vocal sequences emitted from the same bat during low aggression (**A**) and medium aggression (**B**) contexts. All syllables were classified as DFMs. The medium aggression bout lasts for an additional 2.1 s. (**C**) Example high aggression bout from a different bat, with syllable classifications above each waveform. Note that the rBNBl syllable includes a bifurcation of torus near the beginning of the syllable. Peak amplitude of the entire bout was normalized.

In the *tactile irritation paradigm*, a roosting bat received mild tactile stimulation by an experimenter manipulating a cotton swab. This mimicked the natural jostling that occurs among roosting bats, while allowing the experimenter to control the duration of the irritation. While the tactile stimulation often elicited aggressive displays and vocalizations, bats typically did not try to retreat from the cotton swab. For heart rate monitoring trials, tactile stimulation was delivered at a rate of ∼1/s for short (≤15 s) or long (60 s) durations.

**Figure 5:**
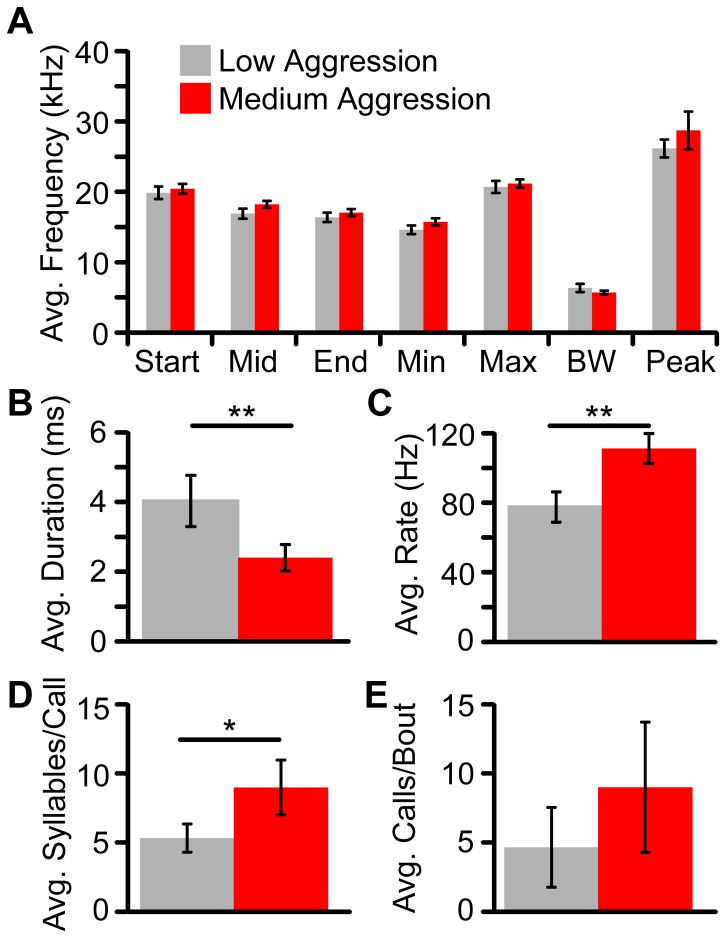
Medium aggression reveals a distinct call structure. (**A**) DFMs syllables do not show spectral differences between low aggression (*grey*) or medium aggression (*red*) contexts. However, DFMs syllable duration was significantly shorter (**B**), syllables were emitted at a faster repetition rate (**C**), and there were more syllables per call (**D**) during medium aggression. Although not significantly different, the number of calls/bout tended to be greater during medium aggression bouts (**E**). Bars represent mean ±2 standard errors. Asterisk, *p*<0.01. Double asterisk, *p*<0.001.

Because intruder bats typically did not vocalize as they approached a roosting cluster of home-cage bats, vocalizations recorded during aggressive contexts rarely contained overlapping signals from multiple animals. In most aggressive encounters the caller could be visually identified by having an open mouth that was directed toward the intruder. However, biosonar vocalizations were more likely to overlap appeasement vocalizations, as the latter were typically emitted while another bat was crawling and exploring the cage with biosonar calls. Because bats did not show overt (visible) signs while emitting appeasement vocalizations, the identity of the caller could not always be determined. In trials with only two bats in a cage, the individual emitting appeasement vocalizations could sometimes be identified by ruling out the bat that was concurrently emitting biosonar signals, which are accompanied by visible, stereotyped behaviors.

**Figure 6:**
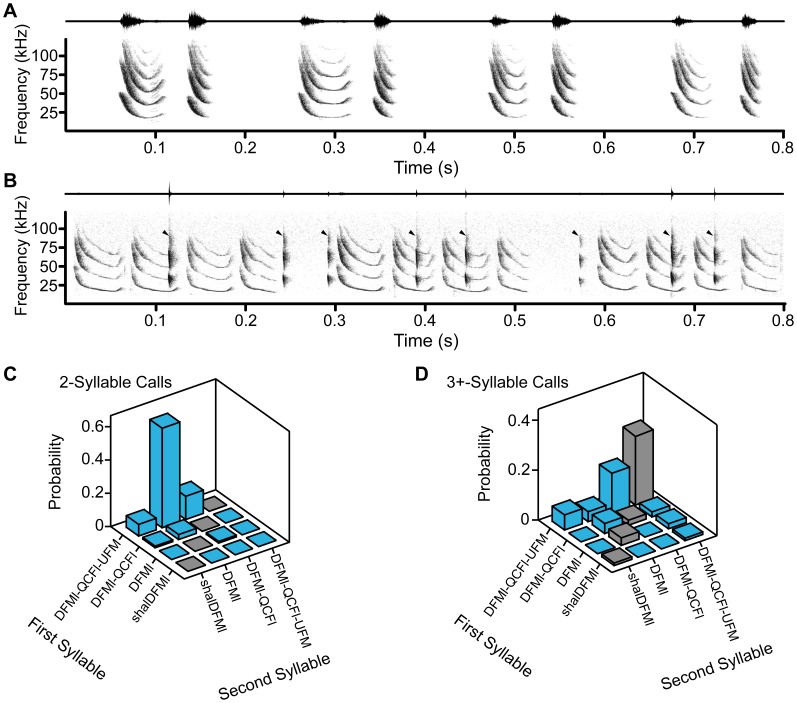
Two distinct appeasement call structures. Example waveforms (*upper trace*) and spectrograms (*lower trace*) showing two distinct call sequences. (**A**) A two-syllable call sequence that transitions from DFMl-QCFl-UFM to DFMl. (**B**). A three or more syllable call sequence that repeats DFMl-QCFl-UFM three times and then transitions to DFMl-QCFl at the end of the call. This calls sequence also shows overlapping biosonar calls (*arrowhead*) emitted by another bat. Peak amplitude of the entire bout was normalized. Due to the large difference in amplitude between the biosonar and appeasement syllables, most appeasement syllables are not visible in the waveform trace shown in B. Examining the probability of two-syllable pairings reveals distinct syllable ordering rules for appeasement calls that contain only two syllables (**C**) or those with at least three syllables (**D**). *Blue bars* represent syllable transitions and *grey bars* represent repeated syllable pairings.

Echolocation (biosonar) signals of *E. fuscus* are highly stereotyped and consist of short duration (2–5 ms) downward frequency modulated sweeps, with a fundamental acoustic element sweeping from 50 to 20 kHz and two to three harmonics [8], [32], [37], [38]. The biosonar signals we recorded often overlapped calls from another bat or were emitted at the end of a bout of social vocalizations. Biosonar signals are easily distinguished from adult social vocalizations and were excluded from our analysis.

**Figure 7:**
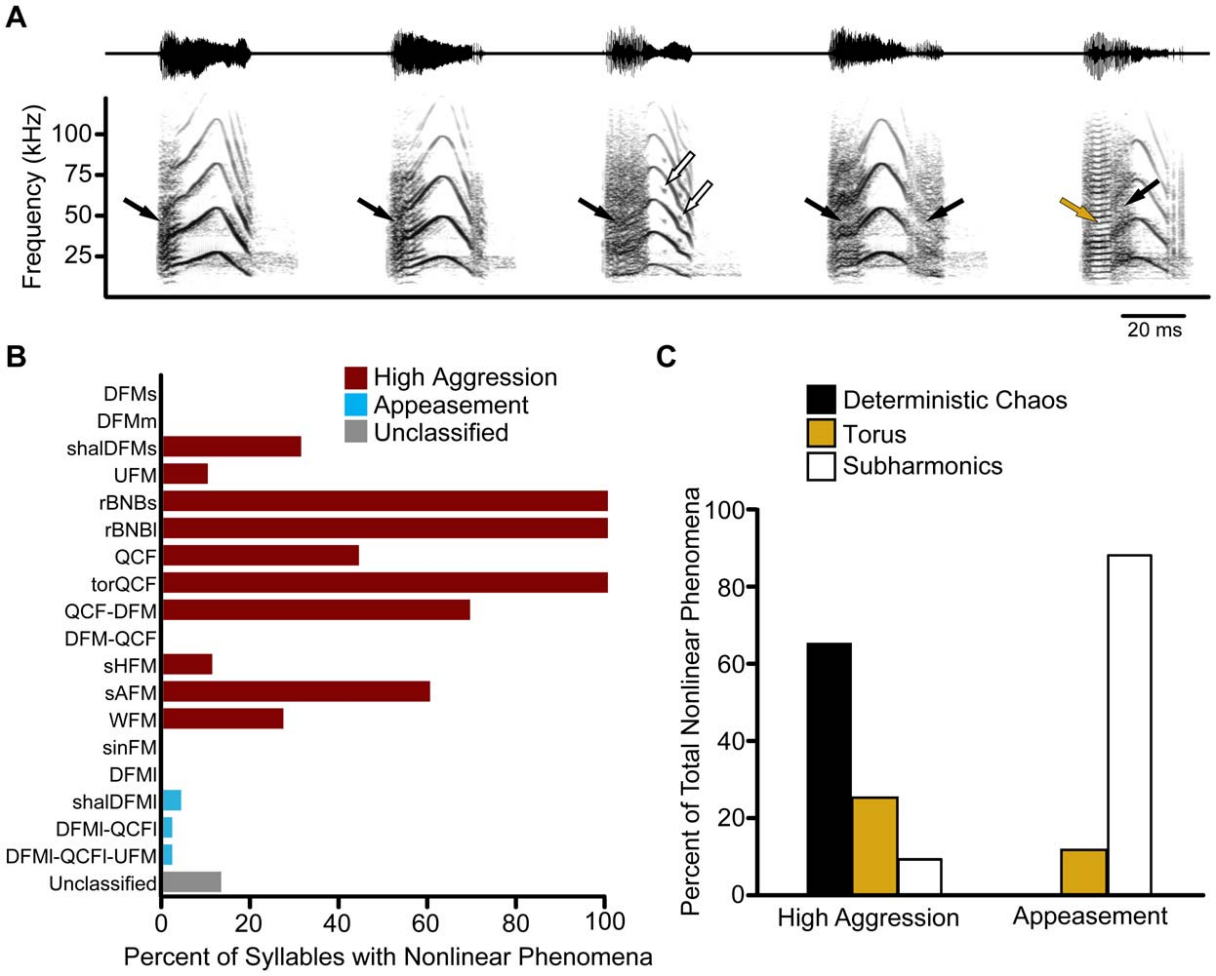
Nonlinear phenomena are differentially observed across syllable type and behavioral context. (**A**) Example waveforms (*upper trace*) and spectrograms (*lower trace*) of sAFM syllables emitted from the same bat. Syllables contain varying degree and type of nonlinearities, such as deterministic chaos (*black arrow*), subharmonics (*open arrow*), and torus (*gold arrow*). (**B**) Nonlinear phenomena were more commonly observed in high aggression syllable types. (**C**) Deterministic chaos was the most common nonlinear phenomenon in high aggression syllables. Although less common overall, nonlinearities observed within appeasement syllables were more likely to be of the subharmonic type.

### Analysis of Vocalizations

#### Terminology

We describe the social vocalizations of *E. fuscus* using nomenclature similar to Bohn et al (2008) and Kanwal et al (1994):

A *syllable* is the smallest acoustic unit of a vocalization, defined as one continuous emission surrounded by background noise. Simple syllables consisted of a single sound element: a quasi-constant frequency (QCF), frequency modulated (FM), or noise burst emission. Composite syllables consisted of two or more distinct sound elements. Syllable types were named according to acoustic structure, with a prefix denoting secondary spectral features (e.g. DFM, downward FM; UFM, upward FM) and a suffix denoting secondary temporal features (e.g. s, short; m, medium; l, long). Composite syllables were often named as sequential hyphenated combinations of individual simple elements. For clarity, some syllable names were modified from those previously presented [36].

**Figure 8:**
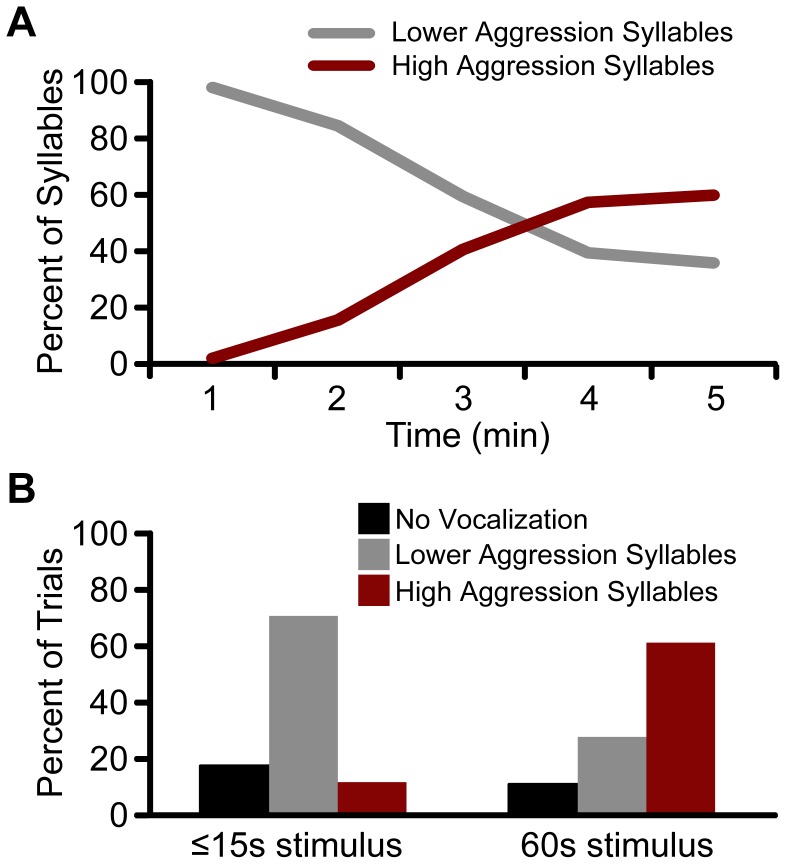
Prolonged tactile stimulation evokes high aggression syllables. (**A**) Example data from a single bat during a 5-min trial of tactile irritation. The bat began emitting lower aggression syllables (i.e. DFMs and DFMm) but later transitioned to high aggression syllables. (**B**) Short duration (≤15 s) tactile stimulation at a rate of ∼1/sec was more likely to elicit lower aggression syllables, whereas a longer duration of stimulation (60 s) evoked high aggression syllables.

A *call* is the simplest emission pattern of a vocalization. Calls were defined by setting inter- and intra-call boundaries (see below), which determined the number of syllables within a call. Calls were composed either of a single syllable (monosyllabic) or groups of two or more syllables (multisyllabic). Simple multisyllabic calls contained the same repeated syllable type, whereas complex multisyllabic calls contained multiple syllable types.

A *bout* is a group of calls relating to a specific behavior or interaction.

**Figure 9:**
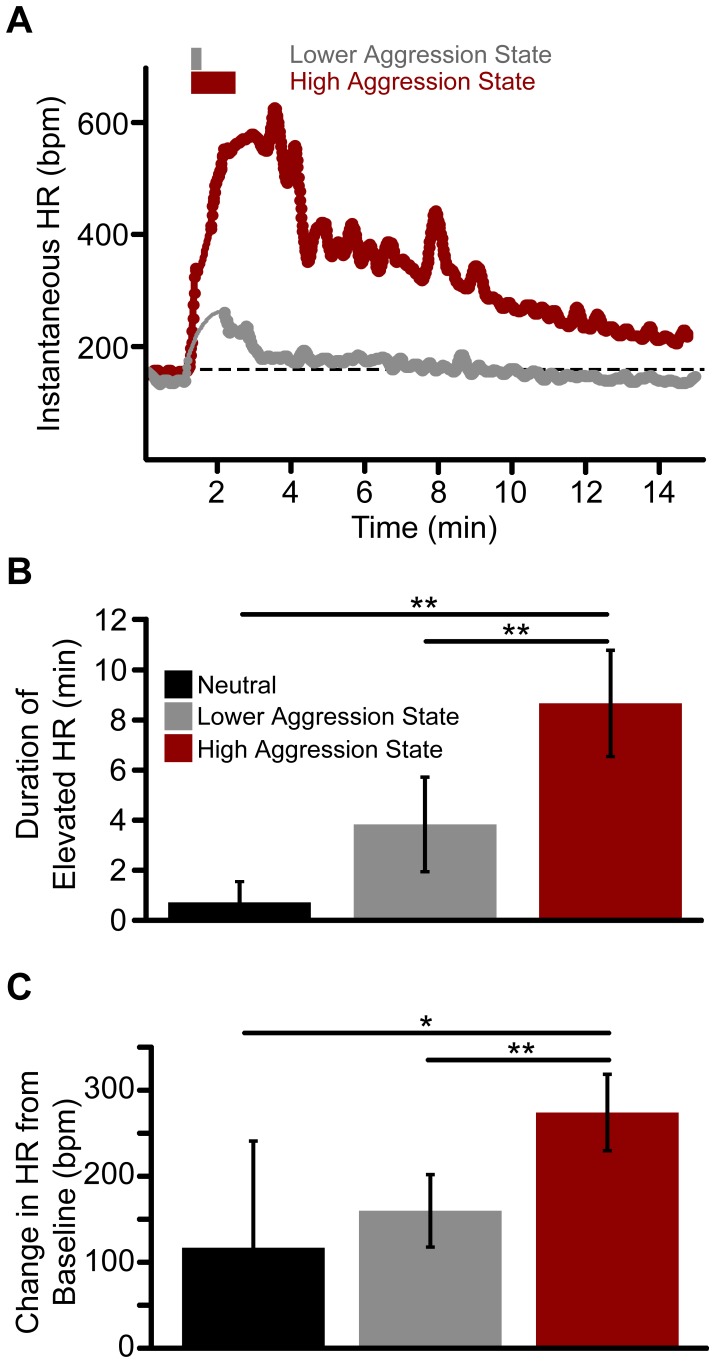
Magnitude and duration of elevated heart rate scales to the level of vocal aggression. (**A**). Instantaneous heart rate (beats per min, bpm) during two 15-min tactile irritation trials from the same bat. A short duration irritating stimulus (15 s) resulted in a lower aggression state (*grey*) and a smaller and shorter increase in heart rate. In contrast, a longer duration (60 s) tactile stimulus resulted in a high aggression state (*red*) and a larger increase in heart rate that remained significantly elevated throughout the trial. Aggressive behavioral states were classified based on the emitted syllable types. *Dashed black line* is the mean baseline rate calculated during the first 60 s of the trial. *Grey and red rectangles* above plot indicate timing and duration of the tactile stimulation. On average, heart rate remained elevated for a longer duration (**B**) and showed a greater magnitude of change (**C**) during high aggression states (*red*) than lower aggression (*grey*) or neutral states (*black*). Bars represent mean ±2 standard errors (n = 39 trials, 4 bats). Asterisk, *p*<0.05. Double asterisk, *p*<0.01.

#### Syllable measurements

Vocalizations were analyzed using SASLab Pro (v5.1, Avisoft Bioacoustics). Syllable start and end times were automatically detected using a standard threshold level (5%) and hold time (1 or 10 ms), and manually adjusted when necessary. Signals were high-pass filtered at 1 kHz and the amplitude of each syllable was normalized to 0.75 V. Temporal analyses were obtained from the sound spectrogram with an FFT length of 256 points (98.43% overlap) that allowed for a temporal resolution of 0.016 ms. Using automated parameter measurements, syllable duration was measured as the end time minus the start time. The inter-syllable silence (ISS) period was defined as the time between the end of one syllable and the start of the next. For spectral analyses we used a FFT length of 512 points (98.43% overlap), allowing for a frequency resolution of 488 Hz. Using automated parameter measurements, we obtained the minimum frequency, maximum frequency, and bandwidth from the total waveform, including all frequencies within 20 dB of the peak spectral amplitude. Peak frequency was measured from the maximum amplitude of the waveform. To accurately measure the fundamental frequency characteristics using automated parameter measurements, syllable harmonics were manually deleted from the waveform. Fifty frequency measurements regularly spaced along the fundamental frequency were obtained. Bandwidth, start, middle, end, minimum, and maximum frequency of the fundamental were computed. Peak-to-peak amplitude was measured to identify syllables that were saturated (overloaded). Saturated syllables were excluded from syllable classifications and all spectral-based analyses, but were included in temporal analyses.

#### Temporal analysis of calls

To classify calls as mono- or multisyllabic, we determined the appropriate inter- and intra-call boundaries. The distribution of all ISS periods was bimodal, with a separation at 35 ms (data not shown). This boundary choice was appropriate across the different syllable types and behavioral contexts. Thus, syllables were categorized as belonging to the same call when the ISS was <35 ms, whereas an ISS ≥35 ms marked the beginning of a new call. All syllables were numbered based on their position within a call, and each call was numbered within a vocalization bout. Inter-call silence (ICS) periods were calculated as the time between the end of one call and the start of the next. Recording files were divided into the appropriate bouts of vocalizations that unambiguously correlated with a specific behavioral interaction. A new bout was defined in cases where a silent period of >1560 ms (2 SD above the mean ICS) occurred between vocalizations.

#### Automated classification of syllable types

In a preliminary analysis, 7632 labeled syllables were classified by an experienced observer based on visual inspection of spectrograms, and 19 syllable types were identified as having unique spectral-temporal features. The following rules were adopted to aid classification of syllable types sharing similar features:

1. QCF syllables were quasi-constant frequency with a fundamental frequency bandwidth <2 kHz.
2. DFM syllables were downward frequency modulated sweeps with a fundamental frequency bandwidth >5 kHz.
3. Shallow DFM (shalDFM) syllables were downward frequency modulated sweeps with a fundamental frequency bandwidth ≥2 kHz but ≤5 kHz.
4. Rectangular broadband noise (rBNB) syllables were classified into two groups by their duration; short syllables <50 ms were labeled as rBNBs, and long syllables ≥50 ms as rBNBl.
5. The composite syllable DFMl-QCFl-UFM had an end frequency that was at least 500 Hz greater than the syllable’s minimum frequency; otherwise it was labeled as DFMl-QCFl.

This preliminary analysis enabled us to create appropriate templates for a spectrogram cross-correlation procedure that automated syllable classification and minimized experimenter bias. All analyses reported in the “Results” are based on syllable classifications determined by the automated method. Labeled syllables were automatically classified with a two-dimensional cross-correlation procedure (SASLab Pro, Avisoft Bioacoustics) that allows a set of user-defined spectrogram templates to be compared against labeled sections of a sound file. The comparison is performed by sliding the two spectrograms past each other in the time domain. An additional sliding in the frequency domain is used to tolerate frequency deviations between the spectrogram template and a labeled syllable. Correlation coefficients were computed for all time- and frequency-offsets, and the peak correlation value recorded. To account for signal variation and to improve syllable recognition, multiple templates of each syllable type were included. We randomly selected 15 exemplars as templates for each of the 19 experimenter-classified syllable types, except for three syllable types that had less than 15 observed examples–the DFM-UFM (n = 14), the wrinkled FM (WFM; n = 14), and the sinusoidal FM (sinFM; n = 13) syllables–in which case all observed examples were used as templates. A bandpass filter (5–50 kHz) was used to limit the range of spectral components compared. Maximum frequency deviations of 5 kHz were tolerated. Duration differences up to 15 ms were tolerated, limiting comparison of labeled syllables to templates that were similar in duration. This prevented misclassifications owing to an increased probability of a significant correlation when a longer composite syllable was compared to a shorter duration template sharing a common feature.

The correlation coefficients for each comparison were used to calculate an average correlation for each syllable type. Labeled sections were also compared to templates of background noise, and the average correlation across all syllable types was 0.3. A labeled syllable was scored as “unclassified” when the average correlation for a syllable type was <0.3. To ensure syllables shared acoustic features with an entire syllable category rather than a single exemplar, syllables were classified to the category with the highest average correlation across all templates. Moreover, template syllables were not guaranteed classification to a particular syllable type. For example, one of the less common syllable types, the short duration DFM-UFM, showed a high degree of variation among its template exemplars and labeled syllables were never automatically classified to this syllable type. Thus, although the experimenter classification based on visual inspection of the spectrogram detected 19 distinct syllable types, the automated computer classification only classified 18 syllable types. Computer classified syllables showed 70% correspondence with syllables visually classified by an experimenter, and less than 2% of syllables remained “unclassified”. When discrepancies occurred, they were often between syllable types that shared similar acoustic features, posing similar challenges for the automated and experimenter classifications.

Because files recorded in an appeasement context often had biosonar signals that overlapped the higher harmonics of the appeasement vocalizations, we compared templates to modified appeasement files in which the harmonics (and biosonar signals) were deleted from the waveform. The DFMl-QCFl-UFM syllable remained a challenge for automated classification because it shared acoustic features with other appeasement syllables and the duration of this syllable was not long enough to prevent these template comparisons in order to aid classification. Thus, appeasement syllables were re-classified as DFMl-QCFl-UFM if the end frequency was at least 500 Hz greater than the minimum frequency.

Because the spectrogram cross-correlation procedure is neither designed nor sensitive enough to detect nonlinear phenomena within syllables, we relied on experimenter classification of nonlinear phenomena by visual inspection of spectrograms. When syllables were qualitatively judged to contain nonlinearities, the type and relative position of the nonlinearity within the syllable was noted. Saturated syllables were excluded from the analyses of nonlinear phenomena.

#### Call sequences

To examine the syllable sequencing within multisyllabic calls, we tracked several features of call structure including: call composition (simple or complex), number of syllables per call, two-syllable pairings, first syllable, last syllable, and location of syllable transitions. The probabilities of all possible two-syllable pairings were then computed across different types of call structures. If a saturated syllable interfered with the determination of call composition, the entire call was removed from the call sequencing analysis. We also excluded bouts of vocalization containing >25% saturated or overlapped syllables.

### Electrocardiogram (ECG) Monitoring

#### Surgical procedures

Four bats used for ECG monitoring underwent surgery to attach a head pin that allowed an external heart rate (HR) monitor to connect with a wireless headstage (Triangle BioSystems Inc., TBSI, Durham, NC). Prior to surgery, animals received an intraperitoneal injection of Torbugesic (5 mg/kg, i.p., Fort Dodge Animal Health, Overland Park, KS), then were anesthetized with Isoflurane inhalation (2–4%, Abbott Laboratories, North Chicago, IL). Hair overlying the dorsal surface of the skull was removed with a depilatory lotion. A midline incision was made and the underlying muscle reflected laterally to expose the dorsal surface of the skull. A metal pin with connectors was cemented to the skull. Hair overlying the dorsal flanks on both sides of the back was removed to allow electrodes to contact the skin. Immediately following surgery, local anesthetic (Lidocaine) and antibiotic cream were applied to surgical areas. The bat was returned to its holding cage for a two-day recovery period before physiological recording.

#### ECG recording & analysis

Prior to attaching the HR monitor, animals were lightly anesthetized by Isoflurane inhalation. The ECG was recorded differentially with two silver wires, one pressed against each exposed dorsal flank. A small amount of electroconductive gel (Parker Laboratories Inc., Fairfield, NJ) was applied to each flank, and the wires attached with a square adhesive bandage. Extra wire slack around the head pin allowed for a full-range of head movements. Physiological signals were transmitted to the TBSI receiver via a wireless headstage, permitting the bats to behave freely within the experimental chamber. Most bats did not scratch at or attempt to remove the HR monitor, and would roost and behave normally within 30 minutes of attachment. The combined weight of the wireless headstage and HR monitor was 4.7 g.

Analog output from the receiver was amplified and bandpass filtered (100–500 Hz; Model 3600, A-M Systems, Inc., Sequim, WA), digitized (10 kHz/channel, 12-bit depth; DataWave Technologies Inc., Loveland, CO), and stored on a computer. DataWave software controlled physiological and video data acquisition. A threshold was used to detect the positive deflection of each R-wave within the QRS complex, and timestamps saved.

Bats were allowed 30 minutes to recover from anesthesia, acclimate to the experimental chamber, and achieve a stable baseline HR. The ECG was recorded over 15-min trials, along with video and vocalizations. The first 60 s of a trial was used to monitor the baseline rate. After 60 s, the experimenter entered the chamber and immediately applied tactile irritation with a cotton swab at a stimulation rate of 1/s for either a short duration (≤15 s) or long duration (60 s), and then exited the chamber.

For tactile irritation trials, the proportion of emitted syllable types was used as a measure to classify behavioral state. For these analyses, we considered low and medium aggression syllables (i.e. DFMs and DFMm) together as “lower aggression”. Each trial was scored as evoking either a neutral, lower, or high aggression state. A neutral state was scored when bats did not emit social vocalizations in response to the tactile stimulus. When more than 80% of syllables in a trial were classified as either DFMs or DFMm, the trial was scored as having evoked a lower aggression state; however, when more than 20% of the syllables in a trial were classified to high aggression syllable types, the trial was scored as having evoked a high aggression state.

DataWave files were imported into MATLAB (MathWorks, Natick, MA) for quantitative analyses. Instantaneous HR was computed as the reciprocal of R-R intervals in beats per minute (bpm). Some trials contained significant movement artifacts during the tactical irritation period and the following additional steps were taken to reduce this artifact. Outliers were defined as data points more than two standard deviations (SDs) below the mean baseline rate or greater than 1200 bpm, a value above physiological maximum [39], [40]. After outliers were removed, instantaneous HR values were binned at 1 s, and a 10-s sliding window shifted in 1-s increments, was applied to smooth the data. A shape-preserving piecewise cubic interpolation was applied to fill in missing values from outlier removal.

Baseline HR was calculated as the average instantaneous HR during the first 60 s of a trial. Starting from the end of the tactile stimulation period, the period of elevated HR was calculated as the duration over which HR remained continuously >2 SDs above baseline. In some cases, HR would return to baseline and then show later elevated peaks. The duration of elevated HR was conservatively measured only from the elevated peak containing the time bin with the maximum HR value. Peak HR values likely occurred during the period of tactile irritation when movement artifacts had the potential to obscure accurate measurement. Thus, in order to measure the magnitude of change, the baseline HR was subtracted from the instantaneous HR measured 15 s after the end of the tactile irritation period (1-s bins).

### Statistical Analyses

Data are reported as the mean ± SD unless stated otherwise. Independent samples t-tests were used to compare descriptive statistics of low and medium aggression vocalizations. Chi-square tests were used on categorical variables to compare the likelihood of syllables occurring across the start, middle, and end of a call. A one-way analysis of variance (ANOVA) with a Bonferroni correction was used to compare HR across behavioral states.

## Results

Big brown bats emitted bouts of vocalization that could be assigned to a specific behavioral context: (1) low aggression, (2) medium aggression, (3) high aggression, or (4) appeasement. First, we briefly describe the acoustic features of the different syllable types recorded. For each behavioral context, we then describe the probability of different syllable types occurring, their acoustic structures, temporal emission patterns, and the probability of syllable transitions. Finally, we relate levels of evoked vocal aggression to heart rate, a measure of the vocalizing bat’s internal state.

### Syllable Acoustics

During an initial analysis, 7632 syllables were automatically labeled and computer classified using a spectrogram cross-correlation procedure. Our automated analysis revealed 18 syllable types unique in their spectral-temporal features (Fig. 1) and descriptive statistics (Table S1). Ten of the 18 syllable types were considered simple syllables. Five of the simple syllables were DFM syllable types, each distinct based on a combination of signal bandwidth and duration. Short (DFMs; Fig. 1A), medium (DFMm; Fig. 1A), and long (DFMl; Fig. 1B) DFM syllables were characterized by steep FM and could be discriminated based on duration. Two shallow DFM syllables, shalDFMs (Fig. 1A) and shalDFMl (Fig. 1B), showed slower FM and were distinguished by duration.

The other five simple syllable types (Fig. 1A) included one UFM syllable, two noise burst syllables that were separated based on duration (rBNBs and rBNBl), and two QCF syllable types. The torus QCF (torQCF) syllable was qualitatively distinct due to its dense harmonic stack.

The remaining eight syllable types were composite syllables, named according to their distinct sound components. The QCF-DFM syllable was predominately QCF with a short DFM component at the end, whereas the DFM-QCF syllable began with a short DFM component but was predominately QCF. The single-humped FM (sHFM) syllable was downward FM, with a plateau period or a shallow UFM in the middle of the syllable. The single-arched FM (sAFM) syllable typically had upsweep and downsweep components of similar duration, although these could vary along with the location of maximum frequency. The WFM syllable was defined by having irregular FM and was heterogeneous in bandwidth and duration.

The sinFM syllable displayed regular oscillations of frequency modulation, with the depth and rate of FM varying between syllables. In cases where clear cycles of frequency modulation could be measured, syllable duration varied according to the number of FM cycles; on average, sinFM syllables had 1.8±1.2 FM cycles and lasted 22.4±20.7 ms in duration (range: 1–5 cycles, 9–73 ms duration; n = 13).

The DFMl-QCFl syllable contained a steep initial DFMl component that smoothly transitioned to a QCFl component (Fig. 1B). The DFMl-QCFl-UFM syllable (Fig. 1B) shared the initial two components of the DFMl-QCFl syllable, but was distinctly defined by the presence of a short UFM component at the end of the signal. This syllable type was heterogeneous in the duration of the QCFl component and the bandwidth of the UFM component.

Syllable types were categorized as aggressive or appeasing based on the behavioral context in which they were predominately observed. A syllable type was considered aggressive if at least 80% of the syllable examples were emitted in an aggressive context (Fig. 2). Categorization of syllables generally conformed to Morton’s (1977) motivation-structural rules; aggressive vocalizations tended to be lower-frequency and broadband (harsh), whereas appeasing vocalizations tended to be higher-frequency and more tonal [41]. Although we did not measure sound pressure level of any syllables, appeasement vocalizations were dramatically lower in amplitude than aggressive vocalizations or biosonar pulses. These vocalizations typically required microphones to be set at higher gain (+30 dB), and were often not detected on microphones optimized for recording aggressive and biosonar vocalizations.

We examined the probability of different syllable types occurring across the four behavioral contexts (Fig. 3). The majority of syllable types were relatively unique to a particular behavioral context, with the exception of the DFMs syllable: it was the most common antagonistic vocalization and was emitted during low, medium and high levels of aggression. Both males and females emitted social vocalizations in each of the behavioral contexts.

### Low Aggression Vocalizations

#### Behavioral context

Antagonistic behavior was commonly observed during the jostling or disruption of roosting bats. Low aggressive vocalizations were emitted by roosting bats that were disturbed by another bat attempting to roost nearby. In this context, the caller may direct their head and bare their teeth towards the bat causing the disturbance (Video S1, Video S2).

#### Syllable structure

Low aggression vocalizations consisted almost exclusively of a single syllable type, DFMs (94%, 194 syllables; Figs. 1,3,4). A small percentage of syllables (6%) were classified to other syllable types sharing similar acoustic features (e.g. DFMm; Fig. 3).

#### Call structure

Almost all of the low aggression calls (92%, 44 calls) were multisyllabic. Analysis of syllable sequencing within multisyllabic calls revealed that 69% (18 calls) were simple multisyllabic, repeating the same DFMs syllable. The majority (86%) of low aggression calls started with the DFMs syllable, and when transitions to other syllable types occurred they were equally distributed throughout the start, middle, and end of the call (χ^2^(2, N = 13) = 2.00, *p*>0.05). Low aggression calls typically contained between 4 and 6 syllables (5.3±2.6 syllables/call). Calls had an ISS period of 7.8±2.7 ms, and were rapidly repeated within the call at an average repetition rate of 77.5±22.6 syllables/s. Average call duration was 53.6±23.8 ms.

#### Bout structure

Low aggression bouts showed an average ICS period of 110.8±159.4 ms (n = 27 bouts). On average, the entire bout duration was 1261.4±2275.8 ms and typically consisted of 2 to 8 calls (5.3±8.0 calls/bout).

### Medium Aggression Vocalizations

#### Behavioral context

Similar to the low aggression context, medium aggression vocalizations were emitted by roosting bats after they had been jostled or disturbed by a conspecific. Vocalizations were categorized to a medium level of aggression when the caller was observed to bite the bat causing the disruption (Video S3, Video S4).

#### Syllable structure

The DFMs was the most common type of syllable emitted during medium aggression encounters (90%, 649 syllables; Fig. 3), although some calls contained additional FM syllable types not found in low aggression. Acoustic features of the DFMs syllable were analyzed separately for the low and medium aggressive contexts. There was no significant spectral difference between DFMs syllables emitted in low and medium aggressive contexts (Fig. 5A); however, the DFMs syllable duration was significantly shorter during medium aggression (2.4 vs 4.0 ms; t(33) = 3.867, *p*<0.001; Fig. 5B). Thus, bats appear to be using the same syllable type in both low and medium aggression.

#### Call structure

Almost all medium aggression calls (95%, 86 calls) were multisyllabic and the temporal emission pattern differed from low aggression calls (Figs. 4, 5). Medium aggression calls typically consisted of 7 to 11 syllables (9.0±3.0 syllables/call) and contained significantly more syllables per call than low aggression calls (t(34) = −3.23, *p* = 0.003; Fig. 5D). The ISS period was also significantly shorter for medium aggression calls (6.0±1.2 ms; t(31) = 2.68, *p* = 0.012), and syllables were repeated at a significantly higher repetition rate (111.2±13.2 syllables/s) than low aggression calls (t(34) = −4.21, *p*<0.001; Fig. 5C). Average call duration was 72.5±27.9 ms. Although the majority (59%, 38 calls) of medium aggression calls were simple, multisyllabic calls that repeated the same DFMs syllable, there was an increased likelihood of a complex call occurring, with new syllable types occurring at the end of the call. A small proportion of DFMm syllables could be observed in both low and medium aggression calls. Across the two contexts we compared the probability of the other 16 syllable types occurring at the last syllable of each call. Medium aggression calls were more likely to transition to another syllable type at the end of the call compared to low aggression calls (30% vs 0% of all multisyllabic calls, respectively; χ^2^(1, N = 10) = 9.18, *p*<0.01), with the most common transition being from DFMs to the sinFM syllable.

#### Bout structure

Medium aggression bouts had an average ICS period of 86.8±46.9 ms (n = 9 bouts). On average, the entire bout duration was 2001.7±2423.2 ms, and typically consisted of between 4 and 14 calls (9.1±7.1 calls/bout; Fig. 5E).

### High Aggression Vocalizations

#### Behavioral context

Bats transitioned to a high aggression context if the duration of disturbance was prolonged, or if there were several repeated disturbances. Bats showed signs of higher arousal and agitation as their heads tracked the movements of the disturbing bat. Bats emitting high aggression calls would display antagonistic behaviors–baring of teeth, snapping at conspecifics, darting, and defensive posturing–even before the intruder bat made physical contact with the roosting cluster. This lower threshold for response tended to prevent physical encounters (e.g. biting) between bats (Video S5, Video S6).

#### Syllable structure

During high aggression states, bats emitted fewer DFMs syllables (10%, 77 syllables) and increased their syllable diversity (Fig. 3). The most common syllable was rBNBs, although torQCF, DFMs, and sAFM syllables were frequently emitted.

#### Call structure

High aggression calls could be monosyllabic (68%, 392 calls) or multisyllabic (32%, 184 calls). Multisyllabic calls typically contained between 3 and 4 syllables per call (3.2±1.1 syllables/call), and had an ISS period of 10.7±7.7 ms. Analysis of syllable sequencing revealed that multisyllabic calls were more likely to be complex (91%, 129 calls), beginning with the torQCF or rBNBs syllable. The torQCF syllable was most likely to transition to a rBNBs syllable, whereas the rBNBs syllable was more likely to repeat or transition to the rBNBl syllable.

#### Bout structure

High aggression bouts showed an ICS period of 115.9±54.5 ms (n = 41 bouts). The average duration of an entire bout of high aggression vocalization was 1375.3±1586.0 ms, and typically consisted of between 6 and 11 calls (8.5±8.9 calls/bout).

### Appeasement Vocalizations

#### Behavioral context

Appeasement vocalizations were commonly observed in the natural (undisturbed) recording paradigm and were never associated with aggressive displays. These calls appear to promote social contact between conspecifics, as they were emitted by: (1) Lone roosting bats when unsettled cage-mates circled around the cage. (2) Roosting bats during a period of re-adjustment or jostling in the roost (e.g. change in foot position, during grooming). Although such movements physically disturbed roosting bats, they did not evoke overt aggression. (3) Bats released into a cage after being handled by an experimenter and before attempting to rejoin a roost cluster (Video S7, Video S8).

#### Syllable structure

The majority of appeasement vocalizations (82%, 2120 syllables) were composed of three major syllable types (DFMl-QCFl-UFM, DFMl, and DFMl-QCFl) that were emitted almost exclusively in this behavioral context (Fig. 3).

#### Call structure

Appeasement calls could be monosyllabic (69%, 1378 calls) or multisyllabic (31%, 630 calls). The most probable syllable type for a monosyllabic call was DFMl (49%). Multisyllabic calls typically contained 2 or 3 syllables per call (2.4±0.6 syllables/call), with an ISS of 23.1±7.8 ms. Analysis of syllable sequencing revealed that multisyllabic calls were typically complex (86%, 439 calls), and syllable transitions were significantly more likely to occur at the end of the call rather than the start or middle (55% vs 31% and 15%, respectively; χ^2^(2, N = 227) = 55.36, *p*<0.001). Further analysis revealed two common call structures within the appeasement context that followed distinct syllable-ordering rules. (i) Multisyllabic complex calls were most likely (77%) to start with the DFMl-QCFl-UFM syllable type. (ii) In complex calls only composed of two syllables, the most probable call sequence was a syllable transition from DFMl-QCFl-UFM to DFMl (Fig. 6A,C), with an ISS of 29.8±5.4 ms. In complex multisyllabic calls with at least three syllables, the DFMl-QCFl-UFM syllable typically repeated 2–3 times (2.52±0.65 syllable repeats/call) before transitioning to the DFMl-QCFl syllable at the end of the call (Fig. 6B,D), with a shorter ISS of 13.9±6.7 ms.

#### Bout structure

The average duration of an entire bout of appeasement vocalization was 2137.5±2112.0 ms (n = 135 bouts), and typically contained between 10 and 14 calls (11.9±10.3 calls/bout), with an ICS period of 139.1±80.2 ms. Bouts containing the two-syllable DFMl-QCFl-UFM to DFMl call sequence had an ICS period of 103.7±20.9 ms, whereas bouts of the call sequence that repeated DFMl-QCFl-UFM and then transitioned to DFMl-QCFl had a shorter ICS period of only 73.8±25.4 ms.

### Nonlinear Phenomena

Deterministic chaos, subharmonics, and torus (biphonation) are naturally occurring acoustic nonlinear phenomena that arise from the intrinsic dynamics of the vibrating vocal folds of the larynx, which behave as coupled nonlinear oscillators [42], [43]. Based on a qualitative spectrogram analysis, nonlinearities were commonly observed in bat vocalizations (Fig. 7). Figure 7A illustrates the varying degrees and types of vocal nonlinearities in the sAFM syllable emitted by one bat, and demonstrates the challenge that nonlinear phenomena can pose for syllable classification [42]. Notice that a single syllable can contain multiple bifurcations between normal and nonlinear phonations. Deterministic chaos was evident as episodes of nonrandom noise and may show residual hints of a fundamental frequency. Subharmonics introduced additional spectral components occurring at fractional integer values of the fundamental frequency. Subharmonics were observed in the F_0_/2, F_0_/3 and F_0_/4 form, with period-doubling being the most common. Torus (biphonation) appeared as multiple harmonic stacks of two similar but unrelated fundamental frequencies that followed a QCF frequency contour.

Nonlinear acoustic phenomena were common in big brown bat social vocalizations, but were differentially observed across syllable types and behavioral contexts. Nonlinearities were typically absent in syllables emitted during low and medium aggression states (i.e. DFMs and DFMm). Calls emitted during high aggression were more likely to include syllables with nonlinearities compared to appeasement vocalizations (77% vs 2%, respectively; Fig. 7B). Note that syllable types rBNBs, rBNBl, and torQCF, by definition, contain acoustic nonlinearities; while rBNBs and rBNBl contain deterministic chaos, the torQCF syllable contains torus. When nonlinearities did occur in the appeasement context, they were most likely to be of the subharmonic type, whereas high aggression nonlinearities were more likely to be deterministic chaos (Fig. 7C). This was true even when the three syllable types defined by nonlinearities were excluded from the analysis (data not shown).

### Tactile Irritation Paradigm

To further investigate how bats transition social vocalizations across different levels of aggression, we lightly jostled bats with a cotton swab to prolong the duration of disturbance and elicit aggressive behavior. Both male and female bats typically vocalized in response to this tactile disturbance and displayed aggressive behaviors such as turning their head toward, baring their teeth, and biting at the cotton swab. Effective locations for directing the tactile stimulus were the forearms and feet.

We recorded 1157 syllables from a single bat during five minutes of tactile irritation, and all syllables were computer classified. For the purpose of these analyses, we considered low and medium aggression syllables (i.e. DFMs and DFMm) together as “lower aggression” syllables. The probability of occurrence of these lower aggression syllables was compared to high aggression syllables (all other syllables, except appeasement). Figure 8A illustrates how the proportion of syllable types changed with the duration of tactile irritation. This bat initially produced lower aggression syllables; however, as the tactile irritation continued the bat transitioned to a higher proportion of high aggression syllables. Thus, aggressive behavior and vocalizations emitted during experimenter-provoked tactile irritation were similar to those displayed during natural bat social encounters.

The data in Figure 8A demonstrate that the duration of tactile irritation has the potential to evoke a predictable level of aggression and alter an animal’s internal state. To better understand this relationship, we used a paradigm to evoke aggressive vocalizations in four bats while monitoring heart rate to obtain an objective measure of physiological state. Instantaneous HR was recorded over 15-min trials, concurrently with vocalization and video recordings. An additional 5892 syllables were automatically classified, and the average baseline HR across bats was 313±81 bpm. In response to a short irritating stimulus (≤15 s), bats emitted vocalizations indicating a lower aggression state on 71% of the trials, whereas the longer duration irritating stimulus (60 s) evoked high aggression states on 61% of trials (Fig. 8B). Tactile irritation also evoked a rapid increase in HR that slowly returned to the baseline rate (Fig. 9A). HR remained elevated significantly longer in high aggression states (8.7±4.0 min) than in lower aggression (3.9±4.3 min) or neutral states (0.7±0.9 min; F(2,36) = 9.66, *p*<0.001; Fig. 9B).The magnitude of change in HR was also significantly greater for high aggression (274±85 bpm) compared to lower aggression (160±96 bpm) or neutral states (117±141 bpm; F(2,36) = 7.367, *p* = 0.002; Fig. 9C). These results confirm the behavioral state classifications assessed by vocalizations and behavioral displays, and provide an objective measure of the animal’s internal state.

## Discussion

This study characterized adult social vocalizations of big brown bats emitted under four behavioral contexts: low aggression, medium aggression, high aggression, and appeasement. We found that captive big brown bats possess a diverse vocal repertoire, with 18 distinct syllable types automatically classified by a spectrogram cross-correlation procedure. Moreover, emotion-related acoustic cues are evident within the call structure, both in terms of the syllable composition and temporal emission pattern. For example, the DFMs syllable is produced almost exclusively in low and medium aggression contexts, but is shorter in duration, contains more syllables per call, and is repeated at a faster rate during medium aggression contexts. High aggression vocalizations have a greater diversity of syllable types, with rBNBs being the most common syllable type produced. During appeasement, bats emit four different syllables types and show two common call sequences that follow distinct syllable-ordering rules. Finally, changes in the duration and magnitude of elevated heart rate scale to the level of evoked aggression, confirming state classifications assessed by vocal and behavioral displays. These results reveal a complex acoustic communication system among big brown bats in which acoustic cues and call structure signal the emotional state of a caller.

### Syllable Classification

Automated classification is critical for removing experimenter bias, allowing extensive data sets to be processed relatively quickly, and permitting replication and comparison among studies [44]. A variety of techniques exist for automated classification, including frequency contour comparison [45], neural networks [46], hidden Markov models [47], and spectrogram correlation [44], [48], [49]. Although we experimented with other techniques, we chose spectrogram correlation because it performs well, errors are easier to understand, and large training data sets are not required [44]. Further, classification based on the fundamental frequency contours is not appropriate for bat social vocalizations as these signals often contain noise-like elements. The automated spectrogram cross-correlation method matched manual classification for 70% of the syllables. Further, this method was used to classify a novel data set in our tactile irritation trials.

Like any of the methods listed above, the spectrogram correlation approach has limitations. Discrepancies between automated and manual classification tend to occur between syllable types that share similar acoustic features or lack a clear distinguishing boundary. These are challenging for manual classification as well. With automated analysis, any associated bias or error is at least constant and does not change over time [49]. An example of a challenging analysis is with the DFM-UFM category. The automated analysis never assigned any syllables, including the template exemplars, to the manually classified DFM-UFM category. This indicates that the variability among the DFM-UFM templates was too high, and suggests that the syllable boundary should be reconsidered. However, due to the low number of observations of the DFM-UFM syllable, it was not possible to consider dividing this category into additional syllable types.

Another challenge relates to syllables with nonlinear elements. Currently, the spectrogram cross-correlation procedure is not sensitive enough to detect nonlinear phenomena within syllables. However, we are unaware of other procedures that can automatically classify syllables with and without nonlinear acoustic elements. A further limitation to the spectrogram cross-correlation is that templates of simple, single-feature syllables can be highly correlated with composite syllables that share acoustic features. We overcame this by restricting comparisons to only those templates that fell within a tolerated range of durations, or by reclassifying syllables based on simple descriptive rules. Importantly, our major findings do not depend on whether syllable classifications are based on manual classification by an experienced observer or on the automated spectrogram cross-correlation procedure (data not shown).

### Acoustic Communication in Big Brown Bats

Although studying vocal behavior in the laboratory affords several advantages over field recordings (e.g. microphone placement, low background noise), the conditions did not permit a comprehensive sampling of the repertoire of social vocalizations of adult big brown bats. Notably, we observed no mating behavior in captivity and therefore recorded no associated vocalizations. Similar alterations in social behavior under captive conditions have been reported in other species [50], [51]. However, for the behavioral contexts examined here, we observed no differences in syllable types between animals recorded immediately after capture and those that had been in captivity for at least one year. Similarity in the social vocalizations of captive and wild animals has been demonstrated in other bats [14]. Thus, although it is unlikely that we sampled the complete repertoire, we found that captive big brown bats are highly vocal with at least 18 distinct syllables types.

Big brown bats are typically gregarious, forming modest-sized colonies (<100 individuals) with bats roosting in close contact [40]. Although there is no known overt social structure within roosting clusters of big brown bats, clustering is known to play a crucial role in homeothermy. Thus, bats will adjust their position within the roost and their degree of physical contact according to ambient temperatures, roosting in a tight cluster when it is cold and spreading out when it is hot [52]. Many of the antagonistic social interactions that we observed were evoked by the arrival or change in roost position of a conspecific, suggesting that vocal protests may play a role in maintaining appropriate spacing among individuals. Similar observations have been made for other vespertilionids in the wild [52], [53], [54].

Appeasement vocalizations were not easily evoked, requiring long, undisturbed recording sessions. These vocalizations appear to promote social contact among bats, but playback experiments are necessary to understand the functional implications of appeasement calls. Interestingly, the appeasement syllables we observed are similar to several of the FM syllables produced by pups in the presence of mothers [35] to promote approach and retrieval behaviors [25], [55], [56]. These pup vocalizations may be precursors to adult appeasement vocalizations that serve a similar contact call function by attracting conspecifics to the roost [35], [57]. Future studies should examine the variety of contexts in which pups call to better understand the ontogeny and function(s) of these social vocalizations.

The complex social system of colonial bat species demands diversity in acoustic communication. Although nonhuman primates are generally considered to have a more complex social structure, the hypertrophied auditory system of laryngeally echolocating bats and their reliance on sound for foraging and orientation may permit more sophisticated acoustic communication. Bats are nocturnal and spend large portions of their day roosting in dark places, hence vocalizations may have assumed the primary communicative function that visual gestures provide for other mammals (e.g. facial expressions, raised fur). The present study demonstrates that big brown bats have a rich vocal repertoire, including complex call structures with syllable-ordering rules.

### Emotional Expression in Social Vocalizations

Emotion-related acoustic cues are evident in the social vocalizations of big brown bats. Three intensity levels of aggression were observed and vocalizations revealed unique features in terms of syllable composition and temporal emission pattern. While previous studies have inferred internal state from the intensity of behavioral displays, the present study associated vocal structure to objective measures of internal physiological states by recording heart rate from freely behaving and vocalizing animals. We found that big brown bats express emotional state by using context-specific syllable types or by varying the temporal call structure (e.g. syllable repetition rate and number of syllables per call). Such acoustic parameter changes are widespread across mammals, suggesting common coding rules that function to influence the behavior of conspecifics: bats [9], [25], [29], [58], primates [24], [59], [60], tree shrews [27], ground squirrels [61], [62], dogs [28], [63], African elephants [64], and guinea pigs [65]. Moreover, particular sound qualities such as rapid signal onset, short duration click-like energy pulses, upward FM sweeps, rapid amplitude modulations, and noisy frequency spectra routinely alter the attention, arousal, and affect of listeners [66].

Nonlinear phenomena are common in adult big brown bat social vocalizations, and likely contribute acoustic qualities that influence the attention and behavioral state of listeners. Nonlinear phenomena are common in nonhuman vocal repertoires, and likely play a crucial communicative role [43]. Some have suggested that nonlinearities increase individual recognition, dishabituation, and auditory salience, and may also provide cues about caller fitness [42], [66], [67]. In the big brown bat, most nonlinearities occur in vocalizations used in highly aggressive behavioral contexts. Some vocalizations, such as the single-arched FM syllable, display a range of nonlinear content, consistent with the graded signaling of emotional state observed in the “coo” call of rhesus macaques [42]. Other syllables emitted in highly aggressive states (rBNBs, rBNBl and torQCF) are defined by the presence of nonlinearities. This study demonstrates that nonlinear phenomena are common in adult big brown bat social vocalizations and likely influence the behavioral state of listeners.

The present study further relates call structure to the heart rate of a vocalizing animal as an objective measure of internal state. In response to an irritating stimulus, a caller shows a corresponding increase in the magnitude and duration of elevated heart rate that scales with the intensity of the emotional state as indicated by emitted vocalizations. Moreover, internal state changes appear to persist on a longer time scale than the physical stimulus or duration of the overt behavioral response. For example, aggressive vocalizations and behavioral displays ceased once tactile stimulation stopped, but heart rate remained elevated for several minutes.

### Neural Processing of Emotional Vocalizations

A major finding is that vocal signals carry information about an animal’s internal emotional state, specifically signaling the behavioral context (aggression vs appeasement) and level of aggression. Given that one purpose of communication is to influence the behavior of listeners, it is reasonable to expect that acoustic signals will activate brain centers of emotional expression and influence internal physiological state. The amygdala may play a special role, since it participates in evaluating the biological significance and salience of incoming sensory stimuli and orchestrates appropriate behavioral responses [68]. If acoustic features of vocalizations signal affective state of the caller and modify the behavior of the listener, then the amygdala may be particularly responsive to these acoustic features. We have shown that amygdalar neurons in the big brown bat discriminate among social vocalizations by modulating their firing rate and response duration [36]. At the single neuron level, responses were closely related to the behavioral context of acoustic signals, with persistent spike discharge more common in response to aggressive vocalizations. Moreover, dramatic differences in spiking were observed after altering emotion-related acoustic cues, such as increasing the number of cycles in a sinFM syllable, or playing a complex multisyllabic vocalization. The present study provides a more detailed understanding of how call structure expresses the emotional state of a caller, and provides the necessary tools for understanding how acoustic context modulates neural responses to social vocalizations in the amygdala and other brain regions.

## Supporting Information

Table S1 **Descriptive statistics of syllable types emitted by big brown bats.**
(PDF)

Video S1 **Low aggression interaction.** Video clip starts 15 s into the trial. An intruder bat attempts to roost with the cluster, causing a disturbance. The caller directs its head towards the intruder bat while vocalizing.
(WMV)

Video S2 **Low aggression vocalization.** A scrolling spectrogram and waveform (Avisoft Bioacoustics) displays a 5-s segment of the vocalizations produced in Video S1. Playback is slowed to a sampling rate of 44.1 kHz, by a factor of 5.7.
(WMV)

Video S3 **Medium aggression interaction.** Another trial in which the same intruder bat as in Video S1 attempts to roost with the cluster. Video clip starts 8 s into the trial. The caller (white band on both forearms) bites the intruder twice while vocalizing. Both the intruder and another bat (red band on both forearms) emit ultrasonic biosonar pulses towards the end of the clip.
(WMV)

Video S4 **Medium aggression vocalization.** A scrolling spectrogram and waveform (Avisoft Bioacoustics) displays a 5-s segment of the vocalizations produced in Video S3. This medium aggression bout includes several complex multisyllabic calls, with transitions to the sinFM observed at the end of calls. Note that some of the syllables are saturated and show signs of aliasing. Playback is slowed to a sampling rate of 44.1 kHz.
(WMV)

Video S5 **High aggression interaction.** Video clip starts 225 s into the trial, and the intruder bat has already made two failed attempts at roosting with the cluster. The unbanded bat shows signs of higher arousal and agitation by tracking the movement of the intruder, taking defensive postures, and vocalizing before the intruder makes physical contact. At 21 s into the video clip, an additional 97 s of the trial are cut, in which the intruder is roosting alone on the opposite side of the cage. The intruder emits biosonar calls between the aggressive interactions. Note how the other two roosting bats are unresponsive, even during the prolonged high aggression interaction.
(WMV)

Video S6 **High aggression vocalization.** A scrolling spectrogram and waveform (Avisoft Bioacoustics) displays a 5-s segment of vocalizations produced 40 s into Video S5. Playback is slowed to a sampling rate of 44.1 kHz.
(WMV)

Video S7 **Appeasement interaction.** Two bats were placed in the cage for undisturbed recording. Video clip starts 114 s into the trial, in which one bat has been crawling (circling) around the cage producing biosonar calls. The lone roosting bat produces ultrasonic appeasement calls (see Video S8), and the unsettled bat returns to roost together. There is no audio associated with this video.
(WMV)

Video S8 **Appeasement vocalization.** A scrolling spectrogram and waveform (Avisoft Bioacoustics) to visualize a segment of vocalizations emitted during the 5-s prior to the bats roosting together in Video S7. The lone roosting bat emits low-intensity appeasement vocalizations, whereas the unsettled bat produces overlapping high-intensity biosonar pulses. The biosonar pulses were saturated due to the high gain of this microphone, and were not included in any analyses. Note that the high gain level also records shuffling movement noise of the bat (appears as low-intensity broadband noise), as well nail clicks against the cage mesh (appears as low-intensity, short broadband clicks), that are not vocal signals. Playback is slowed to a sampling rate of 44.1 kHz.
(WMV)
