# Supplementary material for: Social Vocalizations of Big Brown Bats Vary with Behavioral Context
Source: PLoS One. 2012 Sep 7;7(9):e44550. doi: 10.1371/journal.pone.0044550 (PMC3436781; doi:10.1371/journal.pone.0044550)
Supplement: Table S1 — Descriptive statistics of syllable types emitted by big brown bats. (PDF) [file pone.0044550.s001.pdf]

Table S1. Descriptive statistics of syllable types

	N Syllables	Syllable Type	Duration (ms)	Peak Frequency	Total Min.Freq.	Total Max.Freq.	Total Bandwidth	F_0 Start	F_0 Middle	F_0 End	F_0 Minimum	F_0 Maximum	F_0 Bandwidth
**Aggression Syllables**													
DFMs	1961	simple	3.19 ± 1.79	26.9 ± 14.7	12.5 ± 3.9	76.7 ± 16.7	64.2 ± 17.1	19.1 ± 6.7	17.5 ± 6.3	16.9 ± 6.6	14.7 ± 3.9	21.5 ± 9.8	6.9 ± 9.0
DFMm	305	simple	9.32 ± 6.73	26.3 ± 9.6	19.2 ± 4.5	73.8 ± 16.6	54.6 ± 16.4	26.5 ± 7.4	20.8 ± 5.8	18.8 ± 5.1	17.8 ± 4.4	27.7 ± 7.8	9.9 ± 7.3
shalDFMs	229	simple	11.72 ± 7.18	25.5 ± 12.5	15.6 ± 3.1	70.6 ± 14.2	55.0 ± 13.9	19.6 ± 6.5	16.9 ± 4.5	15.9 ± 4.6	14.4 ± 2.2	22.5 ± 9.5	8.1 ± 9.5
UFM	77	simple	18.15 ± 4.49	24.0 ± 8.7	17.5 ± 3.5	61.5 ± 16.7	44.0 ± 17.7	18.9 ± 4.6	22.9 ± 6.7	21.2 ± 5.0	16.6 ± 2.1	28.3 ± 8.7	11.7 ± 9.0
rBNBs	730	simple	15.58 ± 4.98	21.4 ± 8.2	11.0 ± 2.4	70.9 ± 11.4	60.0 ± 12.4						
rBNBl	225	simple	85.67 ± 46.36	20.0 ± 7.3	11.3 ± 2.3	66.9 ± 13.4	55.7 ± 14.2						
QCF	65	simple	11.15 ± 8.00	16.8 ± 9.2	12.8 ± 3.4	72.8 ± 15.5	59.9 ± 15.3	16.2 ± 12.5	14.6 ± 6.8	16.1 ± 13.3	12.9 ± 3.6	21.1 ± 17.5	8.2 ± 15.5
torQCF	298	simple	17.17 ± 4.51	20.7 ± 9.5	11.6 ± 2.6	68.9 ± 12.5	57.3 ± 13.2						
QCF-DFM	34	composite	16.68 ± 5.29	26.1 ± 13.6	17.8 ± 3.4	73.0 ± 14.6	55.2 ± 15.2	18.4 ± 3.5	17.4 ± 1.6	15.1 ± 1.7	14.9 ± 1.6	18.7 ± 3.5	3.8 ± 3.3
DFM-QCF	22	composite	11.50 ± 6.55	19.1 ± 3.9	15.3 ± 3.2	64.9 ± 11.3	49.7 ± 11.9	22.1 ± 7.4	17.1 ± 3.3	18.9 ± 7.4	15.5 ± 2.5	28.5 ± 12.4	13.0 ± 11.8
sHFM	43	composite	21.66 ± 13.52	27.9 ± 11.3	18.3 ± 4.1	76.5 ± 16.5	58.2 ± 16.6	22.3 ± 6.6	19.0 ± 4.8	16.9 ± 2.7	16.3 ± 2.5	24.0 ± 7.8	7.6 ± 7.8
sAFM	279	composite	17.74 ± 4.55	23.8 ± 10.0	16.2 ± 2.7	67.9 ± 13.9	51.7 ± 14.1	17.1 ± 6.0	18.2 ± 4.6	16.0 ± 4.9	14.1 ± 2.0	22.2 ± 9.6	8.1 ± 9.5
WFM	99	composite	17.23 ± 19.07	26.7 ± 10.4	18.1 ± 5.9	66.8 ± 17.4	48.7 ± 18.8	20.6 ± 6.1	20.1 ± 6.0	19.8 ± 6.4	17.7 ± 5.0	22.9 ± 7.8	5.2 ± 5.6
sinFM	29	composite	39.71 ± 21.34	26.1 ± 14.1	16.6 ± 5.1	67.3 ± 20.6	50.7 ± 21.0	22.9 ± 15.4	21.4 ± 7.4	18.8 ± 10.1	13.5 ± 4.8	33.0 ± 17.5	19.5 ± 13.5
**Appeasement Syllables**													
DFMl	967	simple	21.11 ± 7.33	29.7 ± 6.9	27.4 ± 5.9	84.2 ± 15.1	56.8 ± 15.1	45.1 ± 7.3	25.9 ± 4.6	20.8 ± 3.3	20.7 ± 3.3	45.3 ± 7.3	24.6 ± 7.4
shalDFMl	292	simple	33.48 ± 16.43	19.3 ± 8.0	17.8 ± 6.1	75.2 ± 29.0	57.4 ± 30.4	24.3 ± 5.8	17.9 ± 3.6	15.7 ± 4.6	15.0 ± 2.9	25.4 ± 7.2	10.4 ± 7.5
DFMl-QCFl	546	composite	38.05 ± 17.61	23.2 ± 6.4	22.4 ± 6.4	77.7 ± 20.9	55.3 ± 21.4	36.3 ± 7.8	19.8 ± 2.9	17.1 ± 3.4	16.8 ± 3.1	36.7 ± 7.7	19.9 ± 7.7
DFMl-QCFl-UFM	860	composite	49.82 ± 18.03	25.1 ± 8.2	24.3 ± 7.0	79.6 ± 20.1	55.3 ± 19.0	40.7 ± 9.5	19.2 ± 3.7	20.1 ± 3.1	16.9 ± 3.1	41.1 ± 9.3	24.2 ± 8.3

Values are mean ± SD. Frequency measurements are in kHz.Blank spaces indicate that fundamental frequencies could not be measured.

F_0 = fundamental frequency
